# Can Systems Thinking Become "The Way We Do Things?" Comment on "What Can Policy-Makers Get Out of Systems Thinking? Policy Partners’ Experiences of a Systems-Focused Research Collaboration in Preventive Health"

**DOI:** 10.34172/ijhpm.2020.70

**Published:** 2020-05-11

**Authors:** Bev J. Holmes

**Affiliations:** ^1^Michael Smith Foundation for Health Research, Vancouver, BC, Canada.; ^2^Simon Fraser University, Burnaby, BC, Canada.; ^3^University of British Columbia, Vancouver, BC, Canada.

**Keywords:** Systems Thinking, Research Co-Production, Knowledge Translation

## Abstract

In "What Can Policy-Makers Get Out of Systems Thinking? Policy Partners’ Experiences of a Systems-Focused Research Collaboration in Preventive Health," Haynes et al glean two important insights from the policy-makers they interview. First: active promotion of systems thinking may work against its champions. Haynes and colleagues’ findings support a backgrounding of systems thinking; more important for policy-makers than understanding the finer details of systems thinking is working in situations of mutual learning and shared expertise. Second: coproduction may be getting short shrift in prevention research. Most participant comments were not about systems thinking, but about the benefits of working across sectors. Operationalizing the ‘co’ in co-production is not easy, but it may be where the pay-off will be for prevention researchers, who must understand the critical success factors of co-production and its potential pitfalls, to capitalize on its significant opportunities.

Many researchers, practitioners and policy-makers celebrated the launch of the Australian Prevention Partnership Centre in 2013 and have followed its progress since. The centre’s stated approach to prevention research (for example, valuing evidence from practice as much as from science) was admirable. Its up-front commitment to ongoing learning and adaptation – demonstrated in several publications over the last five years^1-3^ – was refreshing. Most exciting, to those who use a complexity lens in their work, was the centre’s systems thinking orientation.

In “*What Can Policy-Makers Get Out of Systems Thinking? Policy Partners’ Experiences of a Systems-Focused Research Collaboration in Preventive Health*,”^4^ Haynes et al report on interviews with policy-makers involved with the Partnership Centre. The interviews were part of a mixed methods evaluation of the centre, and their findings – discussed in this well-written, actionable article – will no doubt enable the Partnership Centre to refine its work, as well as help others doing systems-focused prevention research.

It would be a mistake to see only the practical merits of this article, though. At a deeper level I think it contains two important messages worth heeding. The first is that the active promotion of systems thinking may be working against its champions. The second is that co-production – often critical to the creation of robust, implementable evidence – may be getting short shrift in the prevention research world, and beyond. I explore each of these below.

## Systems Thinking: Have We Created a Monster?

As a knowledge translation (KT) practitioner and researcher, I was a vocal advocate of that field and a staunch defender and user of its terms and definitions – until a health promotion colleague said to me a few years ago: “I’ve just learned what KT is; turns out I’ve been doing it for 30 years.” The promotion of KT as new, different, complicated and even rarefied had confused her.

I still believe the emergence of KT in Canadian health research was critical. It named and brought focus to an important set of activities related to evidence use that was previously largely overlooked. Certainly, no one intended to promote KT as out-of-reach or esoteric. But increasingly, when I see KT research thriving often in isolation from the very practices it is meant to improve, I wonder if we have created a monster.

I had the same feeling reading this article, as the authors may have had in analyzing their data. They note that “some people have been doing systems thinking things always, but they haven’t necessarily had the systems thinking labels” (p. 71). Among the participants’ comments: “Is this new? I think it’s important and I agree with it but I feel like I’ve been doing systems thinking for my whole career…” (p. 69).

Among participants, systems thinking triggered “polarised, passionate and ambivalent views” (p. 69). But my read is that no participant argued against seeing problems as complex and potential solutions as multi-faceted. Indeed, there is increasing acknowledgement among many different stakeholder groups that using evidence to improve policy and practice is not possible without addressing the complexity of the systems in which we work.^5^

Rather, the comments from participants seemed more to do with how language and concepts were getting in the way of solving problems. The authors tell us some participants “had little interest in systems thinking per se but saw value in the more concrete methods and tools that it was producing” (p. 72). Participants themselves talked about “painful discussions” and “evangelical researchers,” (p. 69) and becoming “confused and hopeless in systems theory” (p. 70). One interviewee suggests the centre should examine how it communicates its systems orientation.

These comments confirm what I explored at a high level in another piece^6^:that perhaps those of us promoting systems thinking are alienating the people who could most benefit from its approach. To what extent do policy-makers need to understand the field of systems thinking as opposed to learning about the complex problems they are trying to solve, and exploring how they might solve them working in partnership with researchers? What are the “must have” system thinking capabilities and competencies for participation in a research project that employs these techniques?

I was pleased to see that on the Partnership Centre website, systems thinking does not feature large. The focus is on the centre’s purpose: strengthening the evidence base for chronic disease prevention, creating and disseminating knowledge, and building capacity to make more informed choices about prevention. Haynes and colleagues’ findings support this backgrounding of systems thinking, and suggest that more important for policy-makers than understanding the finer details of systems thinking is working in situations of mutual learning and shared expertise.

## So…Is It Really More About Co-Production?

The topic of Haynes and colleagues’ interviews was systems thinking, but according to the authors: “The most enthusiastic accounts of why busy policy-makers were involved with the Centre focused on its facilitation of cross-sector connectivity and collaborative work processes…” (p. 69) and “Their accounts focussed on critical reflection, dialogue with experts, pragmatic capacity building and support, co-production and learning through doing” (p. 72). Those who were not directly partnering on projects, say the authors, found the centre’s outputs less useful.

That participants are embracing the opportunity to work across sectors is encouraging, especially given a large literature over the past two decades on the barriers to successful researcher/policy-maker partnerships because of, in part, the groups’ different world-views, motivations and goals.^7,8^ The positive messages in this study bode well for co-production, defined at its simplest as doing research with those who use it.^9^

Like KT – and like systems thinking, as this article shows – co-production may not appear new or different to people who have been partnering on research for years. For example community-based research, including participatory- and other action-based approaches, is a decades old practice^10^; Van de Ven and Johnson some years ago described engaged scholarship, a collaborative form of inquiry where academics and practitioners leverage their different perspectives and competencies to co-produce knowledge^11^; more recently Graham and colleagues have been advancing integrated KT as a model of research where researchers work with knowledge users.^12^

What does feel new to me, at least in academic health research, is the acknowledgement of inherent potential issues when people get together to co-produce knowledge, and the recognition that these issues must be addressed in order for co-produced research to work for the participants, and to have impact. For years, granting agencies have been launching competitions that require a researcher and research user to partner as principal investigators. These are important opportunities, but there is often an underlying assumption that collaborative granting opportunities in themselves will enable people from very different worlds to partner successfully. Oliver et al^13^ note that much literature focuses on only the positives of co-production; they raise the important notion of “costs” associated with co-produced research: on the research itself, the research process, professional risks for researchers and stakeholders, personal risks for researchers and stakeholders, and risks to the wider cause of scholarship. Boivin and colleagues^14^ note three potential risk areas in co-production: credibility (ie, participants need to learn each other’s language and be seen as valued and relevant sources of knowledge for each other); legitimacy (ie, participants need to be clear on whose behalf they speak and be supported to do so) and power (ie, all participants must be able to influence decisions). I noted earlier the large literature on barriers to researcher-research user partnerships.

It is certainly the case that operationalizing the ‘co’ in co-production is not easy.^15^ But here is where the pay-off will be for the Partnership Centre, as well as for others committed to addressing complex health challenges. In theory – and happily, sometimes in practice, as evidenced by Haynes and colleagues’^14^ interviews – co-production maximizes problem-solving by equitably embracing a diversity of roles, perspectives, experiences and skills, which in turn help make visible the complex context in which problems and their potential solutions exist. This, rather than systems thinking per se, seems to be what is bringing policy-makers to the Partnership Centre, and keeping them there.

So, are those of us working in prevention research paying enough attention to co-production – to understanding its critical success factors and its potential pitfalls, and to capitalizing on its significant opportunities? One of those opportunities might actually be more in-depth knowledge of systems thinking approaches – not as a primary goal, but as the natural result of people working together and understanding each others’ worldviews, expertise, and contributions.

Some more in-depth study of co-production – to understand and support what works for whom and why, under what circumstances – feels timely. The caveat being, of course, that we do not create another monster: that we keep such study close to practice, in an ongoing feedback loop of learning and adaptation, so that research on co-production does not take on a life of its own.

## Summary

A systems perspective is critical if society is to make headway on addressing complex health challenges. The Australian Prevention Partnership Centre should be commended for adopting this perspective as a foundation to their work supporting research collaborations.

However, interviews with participating policy-makers suggest a focus on systems thinking as a “thing apart” – as a discipline or field that needs to be understood over and above the problem and solutions under collaborative study – may not be helpful. The language and concepts of systems thinking may be confusing and even alienating to people who could both benefit from and contribute to important prevention research projects.

Haynes and colleagues’^14^ results point to a strategy of backgrounding systems thinking – ideally, having it become “the way we do things” in deep research collaborations. It may be that we need to know less about how policy-makers engage with systems thinking, and more about how to support them to work in partnership with researchers and others to understand problems, and co-produce solutions.

## Ethical issues

Not applicable.

## Competing interests

Author declares that she has no competing interests.

## Author’s contribution

BJH is the single author of the paper.
